# A New Strategy Involving the Use of Peptides and Graphene Oxide for Fluorescence Turn-on Detection of Proteins

**DOI:** 10.3390/s18020385

**Published:** 2018-01-29

**Authors:** Huan Shi, Bibo Zhang, Shuwen Liu, Chunyan Tan, Ying Tan, Yuyang Jiang

**Affiliations:** 1 Department of Chemistry, Tsinghua University, Beijing 100084, China; shi-h16@mails.tsinghua.edu.cn (H.S.); zbb14@mails.tsinghua.edu.cn (B.Z.); liusw15@mails.tsinghua.edu.cn (S.L.); tancy@sz.tsinghua.edu.cn (C.T.); 2 The State Key Laboratory Breeding Base-Shenzhen Key Laboratory of Chemical Biology, the Graduate School at Shenzhen, Tsinghua University, Shenzhen 518055, China; jiangyy@sz.tsinghua.edu.cn; 3 School of Pharmaceutical Sciences, Tsinghua University, Beijing 100084, China

**Keywords:** peptide, GO, ariginines, sensitivity, selectivity

## Abstract

The detection of proteins is of great biological significance as disease biomarkers in early diagnosis, prognosis tracking and therapeutic evaluation. Thus, we developed a simple, sensitive and universal protein-sensing platform based on peptide and graphene oxide (GO). The design consists of a fluorophore (TAMRA, TAM), a peptide containing eight arginines and peptide ligand that could recognize the target protein, and GO used as a quencher. To demonstrate the feasible use of the sensor for target detection, Bcl-xL was evaluated as the model target. The sensor was proved to be sensitive and applied for the detection of the target proteins in buffer, 2% serum and living cells.

## 1. Introduction

Proteins play key roles in a wide range of human physiological processes, including catalysis, signaling, transport and storage, nutrition, immunity and regulation. The expression of many proteins varies significantly between healthy people and patients with various diseases, which results in pathological changes [1,2]. These proteins are used as disease biomarkers for tumors, neurodegenerative diseases, inflammation and other diseases [3,4,5,6]. It is important to detect disease biomarkers for early diagnosis, prognosis tracking and therapeutic evaluation. Numerous methods have been reported to detect target proteins, such as enzyme-linked immunosorbent assay (ELISA), electrochemical immunology sensors and mass spectra analysis [7,8,9,10,11,12]. However, these methods have some limitations; for example, most involve multistep and time-consuming procedures and often cannot be well applied to intracellular detection. As a result, new strategies for rapid, convenient and sensitive protein detection are needed.

Until now, “turn-on” biosensors involving fluorophore and quencher based on specific recognition have been highly popular [13,14,15,16]. Graphene oxide (GO) has been widely used as a quencher due to its remarkable water-soluble, electronic and mechanical properties. Numerous oxygenic groups throughout the topmost surface layer enable GO to absorb nucleotide acids, amino acids and proteins via electrostatic, hydrophobic, hydrogen bonding and π-π stacking interactions [13,14,15,16,17]. By taking advantage of the strong-yet-selective interactions between peptides and proteins [18], we designed a novel and general strategy involving the use of modified peptides and GO to selectively detect target proteins. Zhang et al. found that GO absorbed amino acids, such as arginine, histidine, lysine, tryptophan, tyrosine and phenylalanine via electrostatic interaction or π-π stacking interaction. The strength of interactions between amino acids and GO is in the following order: Arg > His > Lys > Trp > Tyr > Phe [19]. To simplify covalent modification, we design some arginine residues to the N-terminus of the peptide ligand for efficient absorption by GO in order to develop a general strategy for target detection. These arginine residues, combined with GO via electrostatic interactions between the positively charged arginines and negatively charged edge of water-soluble GO, will enhance the binding ability between GO and the peptide ligand, which further exhibits good specificity to the target protein. This design also makes the whole system more simple and controllable [20,21,22].

In this study, we developed a simple and effective fluorescence “turn-on” protein-sensing platform using peptides and GO (peptide-GO sensor). The platform consists of a fluorophore TAM that modifies the peptide, a peptide containing eight arginine residues and peptide ligand that recognizes the target protein, and GO, which is an excellent quencher of fluorescence molecules [23,24]. The fluorescent peptide binds with GO, giving a fluorescence-off signal because of FRET-based fluorescence quenching and constructing the peptide-GO sensor. In the presence of the target, the turn-on fluorescence changes are monitored as the basis of the detection. To demonstrate the feasibility of using the peptide-GO sensor for protein detection, Bcl-xL, which is a transmembrane molecular protein in the mitochondria considered as a tumor biomarker, was evaluated as a model target [25].

## 2. Materials and Methods

### 2.1. Apparatus and Materials

The fluorescence spectra were acquired on a SPEX Fluorolog 3-TCSPC spectrometer equipped with 1 cm path length cuvettes. The pH value was measured by PHS-3C Precision Ph/Mv Meter. The cell images were acquired on an Olympus Biological Microscope System. The 3 peptides, TAM-PEP (R4): TAM-RRRRNLWAAQRYGRELRRMSDKFVD, TAM-PEP (R6): TAM-RRRRRRNLWAAQRYG RELRRMSDKFVD and TAM-PEP (R8): TAM-RRRRRRRRNLWAAQRYGRELRRMSDKFVD, were synthesized by GL Biochem (Shanghai, China) Ltd. GO (XF-020, 50–200 nm) was purchased from XianFeng Nano Co. (Nanjing, China). The cDNA3.1-Bcl-xL plasmid was constructed by Genecreate Biological Engineering Co. (Wuhan, China) Tris-HCl, IPTG, PMSF, Anti-Bcl-xL antibody, Anti-ACTB antibody, HRP-conjugated Goat Anti-Rabbit IgG, Cell Counting Kit-8, Cytochrome C, BSA, RNase and lysozyme were purchased from Sangon Biotechnology Co. (Shanghai, China) BeyoECL Star and trypsin digestion solution were purchased from Beyotime Biotechnology Co. (Shanghai, China) Lipo ™ 2000 was purchased from Thermo Fisher Scientific Co.(Beijing, China)The deionized water was prepared on a Milli-Q water purification system and used throughout all experiments.

### 2.2. Protein Expression And Purification

The expression and purification of Bcl-xL were carried out according to the previous studies [26,27]. Firstly, the constructed plasmid was transformed into BL21 (DE3) strain of *E. coli.* Secondly, the cells were inoculated into 200 mL 2× YT culture medium added kanamycin (30 μg/mL) with shaking at 37 °C overnight until OD_600_ = 0.6. Next, 1 mM IPTG was added to induce the expression of Bcl-xL for 4.5 h at 25 °C, 220 rmp. After centrifuging at 5000× *g*, 4 °C for 10 min, the precipitation was collected and resuspended in Tris-HCl buffer (50 mM Tris-HCl, pH 8.0, 5000× *g*, 4 °C, 10 min) and added 7 mL lysis buffer (20 mM Tris-HCl, pH 8.0, 500 mM NaCl and 10% glycerol) with lysozyme (0.2 mg/mL) and PMSF (0.5 mM). The cells were sonicated for 30 min and centrifuged at 12,000× *g* for 30 min at 4 °C. The supernatant was incubated on the Ni^2+^-NTA column for 1.5 h, and the His60 Ni Superflow Resin was washed by washing buffer (50 mM Tris-HCl, pH 7.0, 100 mM NaCl, 25 mM imidazole and 10% glycerol). The Bcl-xL could be collected by washing with elution buffer (50 mM Tris-HCl, pH 7.0, 100 mM NaCl, 200 mM imidazole and 10% glycerol). The protein concentration was determined using the BCA method with bovine serum albumin (BSA) as a standard.

### 2.3. Fabrication of Peptide-GO Sensor and Fluorescence Detection

The appropriate amount of the peptides was weighed and dissolved in DMSO at a concentration of 1 mM and diluted to 100 nM with 50 mM Tris-HCl buffer in 2 mL centrifuge tube. Different concentrations of GO were added into the system and quenched the fluorescence of peptides in different degree. The fluorescence spectra of peptides were performed with the excitation wavelength of 540 nm and the emission wavelength from 565 nm to 700 nm. These elements constructed the peptide-GO sensor to detect target protein. Various concentrations of target Bcl-xL protein were incubated with sensor for 1 h and the turn-on fluorescence changes were tested.

### 2.4. Preparation of Human Serum Samples

The serum was removed from the −20 °C refrigerator and placed in 4 °C refrigerator for about 12 h to dissolve in a relaxed manner. Next, it was transferred to room temperature conditions with regularly shaking to make it fully soluble. 5 mL serum was added into the 50 mL glass bottle and diluted with DMEM medium to 50 mL.

### 2.5. Construction of Bcl-xL Overexpression Cell Line

A common cervical cancer cell-Hela was used in our experiments. DMEM medium containing 10% serum was used for cell culture. 1.5–3 × 10^5^ Hela cells were incubated in cover glass bottom dish with 1.5 mL antibiotic-free medium for 12–16 h before transfection to ensure that the cell turbidity reached 75% at transfection time. After incubating in carbon dioxide incubator for 12–16 h, the transfected samples should be prepared, involving 2 µg plasmids diluted into 100 μL Opti-MEM^®^ I Medium and 4 μL Lipo™ 2000 diluted into 100 μL Opti-MEM^®^ I Medium. The two dilutions were mixed, gently shook up and down and placed at room temperature for 20 min, so that the plasmid DNA could be fully encapsulated by liposomes. The cover glass bottom dish was washed 2–3 times with sterile PBS after the medium was removed. Next, 100 μL mixture and 1 mL complete medium were added to each dish, gently mixed and incubated in a CO_2_ incubator. After transfection for 4–6 h, the medium was changed to reduce the toxicity to the cells.

### 2.6. Western Blotting

12% resolving gel was prepared for proteins separation. After transferred onto a polyvinlidene fluoride membrane (PVDF, 0.22 μm), the membrane was stained by Ponceau red and cut at 35 kDa, then washed 3 times in 1× TBST buffer (1 M Tris-HCl 20 mL, pH 8.0, NaCl 8.8 g, Tween 20 2 mL, ddH_2_O) and blocked in 1× TBST buffer containing 3% BSA for 1 h. The membranes were incubated with anti-Bcl-xL antibody and Anti-ACTB antibody respectively at 4 °C overnight, then washed 3 times. After incubated with HRP-conjugated Goat Anti-Rabbit IgG for 2 h and washed 3 times, the membranes were detected using Gel imaging system.

### 2.7. Cytotoxicity Assay

Hela cells were collected and added to a 96-well plate with 8 × 10^3^ cells per well. The plate was maintained at 37 °C in a 5% CO_2_/95% air incubator overnight. After adding 0, 0.5, 1, 10, 20, 50 and 100 μg/mL GO into the wells, the cells were cultured for 24 h at 37 °C. The cells should be washed 3 times with PBS and cultured for another 4 h after 20 μL 0.5% MTT added into the wells. After the remaining MTT solution was removed, 150 μL DMSO was added into each well and the plate was shocked for 10 min to fully dissolve the formazan crystals. Finally, the absorbance was measured by microplate reader at the excitation of 490 nm.

### 2.8. Cell Imaging

Hela cells and Bcl-xL overexpression cells were imaged by confocal fluorescence microscopy as a control. After respectively co-incubated with the TAM-PEP (R8)/GO mixture for 3 h, Hela cells and Bcl-xL overexpression cells were imaged by confocal fluorescence microscopy at the excitation of 559 nm.

## 3. Results and Discussion

### 3.1. The Principle of the Strategy

As a member of Bcl-2 family, Bcl-xL plays an important role in inhibiting apoptosis by forming heterodimers with pro-apoptotic proteins or directly developing an oligomeric channel on the outer membrane of the mitochondria to maintain the normal state of the mitochondrial membrane when the cells are in stress. It is usually found to be overexpression in tumor cells. As a result, researchers always use Bcl-xL as tumor biomarker in previous diagnosis, prognosis tracking and therapeutic evaluation [25]. A commonly used peptide (NLWAAQRYGRELRRMSDKFVD), which has been reported to bind to Bcl-xL through the BH3 binding domain with a dissociation constant in the nanomolar range [28,29], was selected. To verify the efficiency of the strategy, we designed four-, six-, and eight-arginines to the N-terminus of the peptides, which were modified with TAM. The different number of arginines had varying effects on the electrostatic interaction with GO. The three new peptides, TAM-PEP (R4), TAM-PEP (R6) and TAM-PEP (R8), were synthesized and used in subsequent analysis.

**Scheme 1:**
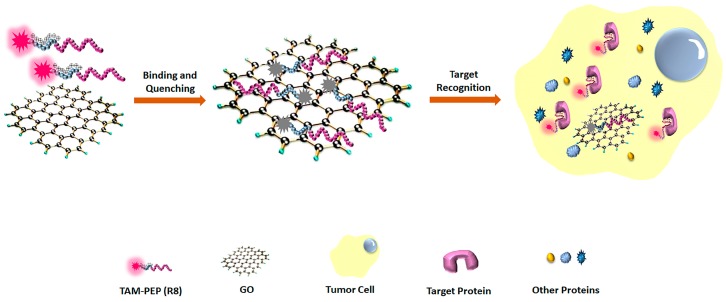
Schematic diagram of turn-on fluorescence assay for target proteins involving the use of peptide-GO sensor.

Scheme 1 shows the principle underlying our strategy. The TAM-modified peptide demonstrated a strong red emission signal (λmax = 580 nm). The fluorescence was quenched by GO when the peptide was absorbed to the surface of GO because of electrostatic interaction. In the presence of the target, the peptide ligand preferentially binds to its target, causing the peptide to separate from the GO surface and turning on the red fluorescence. On the contrary, the peptide-GO sensor showed no fluorescence in the absence of the target. This strategy involving the use of modified peptides and GO was applied for target protein detection in vitro and in cells. In addition, it was found to detect a wide range of target proteins in a sensitive and specific manner by simply replacing the corresponding peptides.

**Figure 1:**
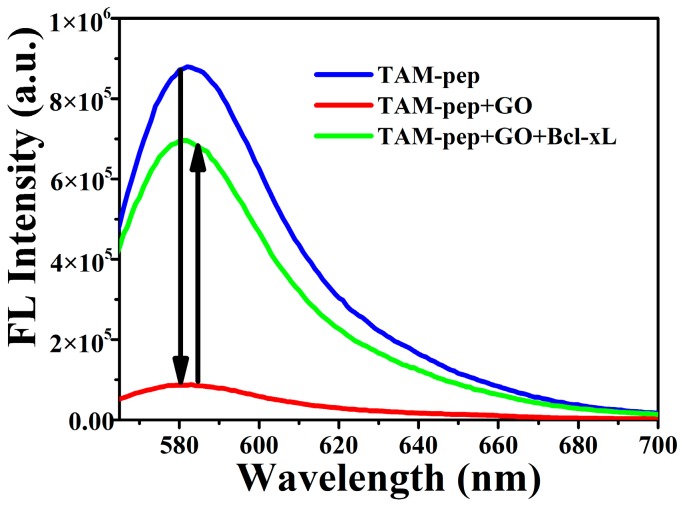
Fluorescence emission spectra of 100 nM TAM-PEP (R8) under different conditions: the fluorescence spectrum of TAM modified peptide (blue line), the fluorescence spectrum of GO-quenched signal (red line), and 5 μM Bcl-xL triggered turn-on fluorescence spectrum (green line). Ex = 540 nm.

To test the fluorescence signal of the peptide-GO sensor with addition of Bcl-xL, 100 nM TAM-PEP (R8) was prepared in 50 mM Tris-HCl buffer (pH 8.0) and irradiated at a wavelength of 540 nm. As shown in Figure 1, a strong fluorescence signal was observed at a wavelength of 580 nm, while the fluorescence was dramatically quenched when 100 ng/mL GO was added to the solution. After adding 5 μM of the target protein (Bcl-xL), approximately 80% fluorescence recovered compared with the original fluorescence of TAM. This result demonstrated that our strategy is feasible for detecting target molecules.

### 3.2. Optimization of the Detection Conditions

**Figure 2:**
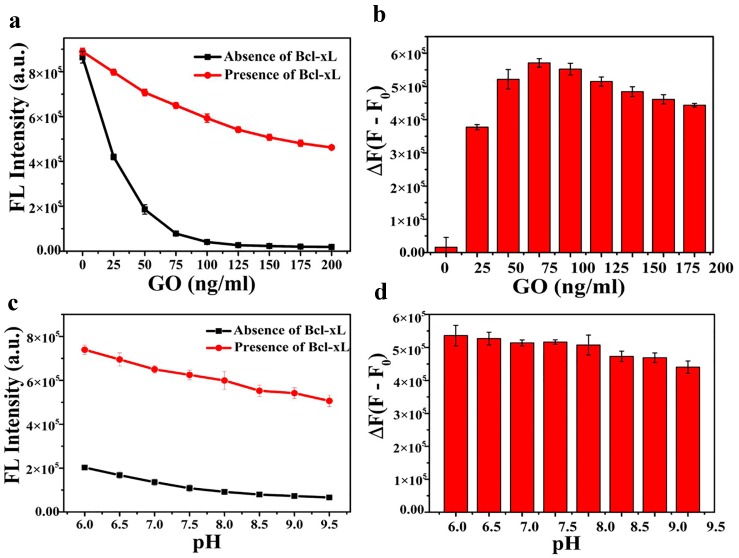
(**a**) Effect of GO concentration on fluorescence intensity of TAM-PEP (R8) at the emission of 580 nm in the absence (black) and in the presence of 5 μM Bcl-xL (red); (**b**) Recovery intensity of fluorescence of TAM-PEP (R8) at the emission of 580 nm with different GO concentrations, where ΔF is the difference of the fluorescence intensity with and without Bcl-xL (5 μM); (**c**) Effect of pH on fluorescence intensity of TAM-PEP (R8) at the emission of 580 nm in the absence (black) and in the presence of 5 μM Bcl-xL (red); (**d**) Recovery intensity of TAM-PEP (R8) at the emission of 580 nm with different GO concentration, where ΔF is the difference of the fluorescence intensity with and without Bcl-xL (5 μM). Experiment conditions: TAM-PEP (R8) 100 nM; Incubation time: 1 h; Room temperature. Excitation: 540 nm.

To make the system highly sensitive and specific, we optimized the experimental conditions including the concentration of GO, pH value of the system and incubation time of the target. The concentration of GO was directly related to the quenching efficiency and affected the interaction strength between the peptide and GO, which was close to the sensitivity value of detection. As shown in Figure 2a, the concentration of GO affected the fluorescence intensity in the absence and presence of 5 μM Bcl-xL. By adding GO to the solution containing TAM-PEP (R8), the fluorescence intensity decreased sharply and reached a plateau at 75 ng/mL in the absence of Bcl-xL. In the presence of 5 μM Bcl-xL, the fluorescence intensity increased to different extents because of the various interaction strengths between the peptide and GO. 

Figure 2b shows that the fluorescence change reached a maximum at 75 ng/mL with increasing concentrations of GO. Therefore, we chose 75 ng/mL GO as the optimal concentration to quench the fluorescence of TAM in Bcl-xL detection, which was much lower than previous studies [16,17,30,31]. Using the same principle, the pH value was optimized over a pH range of 6.0–9.5. There was almost identical change in fluorescence intensity in the absence and presence of 5 μM Bcl-xL with increasing pH. To ensure minimization of the background, pH 8.0 was used in subsequent analysis.

**Figure 3:**
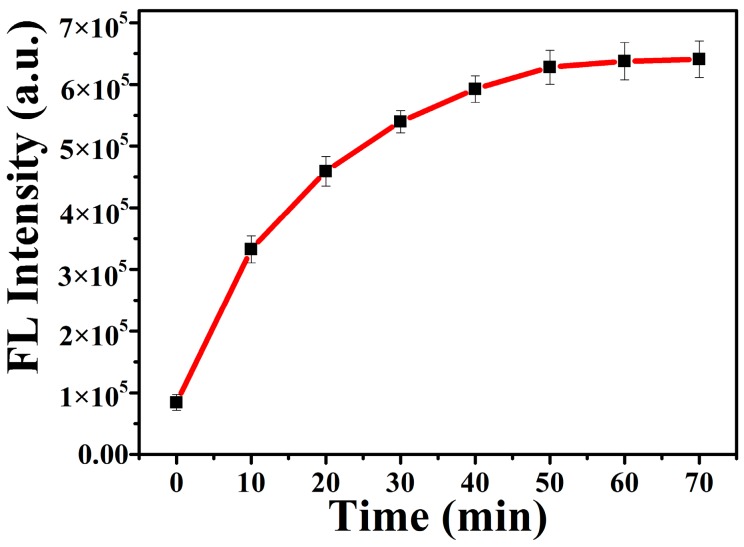
Fluorescence intensity of TAM-PEP (R8) in GO solution by addition of 5 μM Bcl-xL as a function of incubation time. Excitation: 540 nm.

Based on the determined GO concentration and pH value, we studied the response time for the detection of target by the sensor. As can be seen by inspection of the results in Figure 3, addition of 5 μM Bcl-xL caused the intensity of fluorescence of TAM-PEP (R8) to increase gradually, reaching a plateau at 50 min. Therefore, we fixed the response time at 50 min in the detection process.

### 3.3. The Sensitivity and Specificity of the System

**Figure 4:**
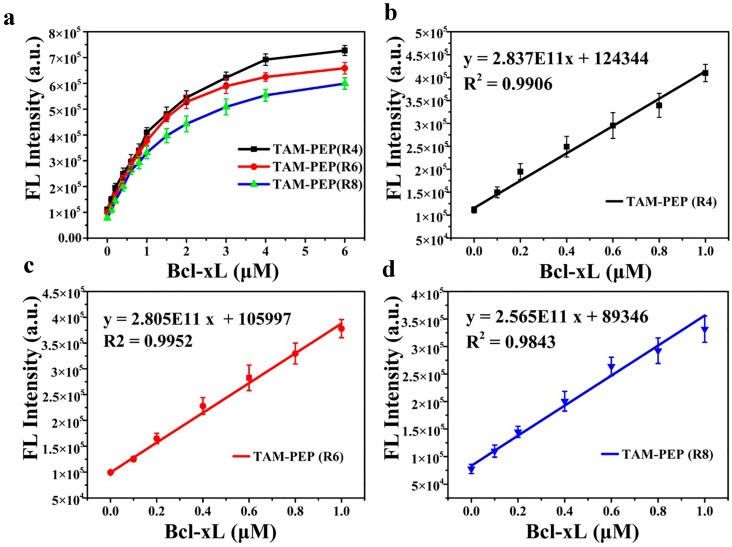
(**a**) Plots of fluorescence intensity of TAM-PEP (R4), TAM-PEP (R6) and TAM-PEP (R8) at 580 nm versus the concentrations of Bcl-xL in Tris-HCl buffer (pH 8.0); (**b**–**d**) Linear relationship between fluorescence intensity and Bcl-xL concentrations.

To optimize the sensitivity and specificity of the assay system, we tested the 3 peptides, TAM-PEP (R4), TAM-PEP (R6), and TAM-PEP (R8). 100 nM TAM-PEP (R4, R6, R8) and 75 ng/mL GO were prepared in 50 mM Tris-HCl (pH 8.0). As shown in Figure 4a, the fluorescence intensity of the 3 peptides markedly increased with the addition of Bcl-xL. The 3 peptides showed nearly the same action at different concentrations of Bcl-xL. Figure 4b–d shows that there was good linear correlation between the fluorescence intensity and Bcl-xL concentrations in the range of 0–1.0 μM, suggesting that Bcl-xL could be quantitatively detected by the sensor. The limits of detection of the three systems were calculated to be 8.4, 9.4, 9.9 nM, respectively, for Bcl-xL using the equation limit of detection = 3 × Sb/S (Figure 4). These results demonstrated that this method could be used for quantitative and sensitive detection of Bcl-xL using the 3 peptide sensors.

**Figure 5:**
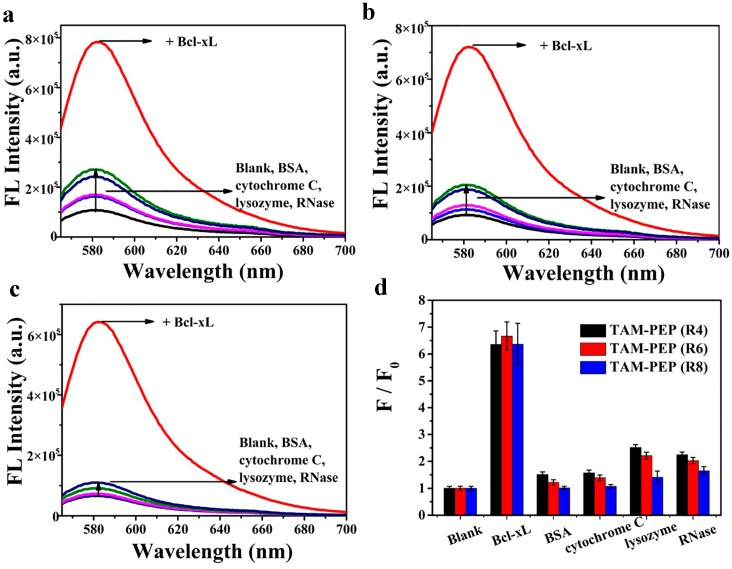
(**a**–**c**) Fluorescence intensity of TAM-PEP (R4), TAM-PEP (R6), TAM-PEP (R8) sensor in the presence of Bcl-xL (5 μM), BSA (10 μM), cytochrome C (10 μM), lysozyme (10 μM), and RNase (10 μM) respectively; (**d**) Fluorescence recovery ratio (F/F_0_) of TAM-PEP (R4), TAM-PEP (R6), TAM-PEP (R8) sensor in the presence of Bcl-xL (5 μM), BSA (10 μM), cytochrome C (10 μM), lysozyme (10 μM), and RNase (10 μM) respectively, where F and F_0_ were the fluorescence intensity with and without proteins.

Specificity is one of the most important factors in evaluating a detection system. Therefore, we investigated the specificity of the 3 peptide sensors. Bovine serum albumin, cytochrome C, lysozyme and RNase were selected and tested at concentration of 10 μM, while the tested concentration of Bcl-xL was 5 μM. The different degrees of fluorescence recovery ratio of the 3 peptides might be due to the varied interaction strengths between the peptides and GO surface. (Figure 5a–c) As shown in Figure 5d, the fluorescence intensity of the three peptides behaved almost identically in the presence of the target proteins, while the fluorescence intensity of TAM-PEP (R8) was obviously lower than TAM-PEP (R4, R6) in the presence of BSA, cytochrome C, lysozyme and RNase. The binding of TAM-PEP (R8) and GO was less disturbed by other proteins compared with TAM-PEP (R4, R6) due to the stronger electrostatic interaction or π-π stacking interaction between more arginines and GO. Considering both sensitivity and specificity, TAM-PEP (R8) was the best peptide-GO sensor for fluorescence turn-on detection of Bcl-xL.

### 3.4. Bcl-xL Detection in Serum

**Figure 6:**
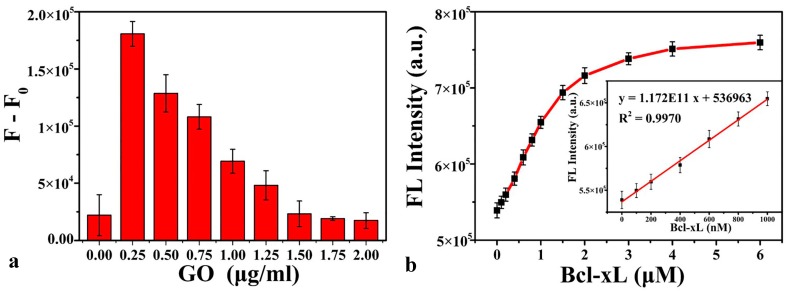
The peptide-GO sensor tested in 2% serum assay. (**a**) The Fluorescence intensity changes of peptide-GO sensor in the presence (F) and in the absence (F_0_) of 5 μM Bcl-xL with the increasing amount of GO from 0 to 2 μg/mL in the 2% serum; (**b**) Fluorescence intensity of TAM-PEP (R8) as the increasing concentrations of Bcl-xL; the inset was the linear relationship between fluorescence intensity and Bcl-xL concentrations in 2% serum assay. Experiment conditions: TAM-PEP (R8) 100 nM; Incubation time: 1 h; Room temperature. Excitation: 540 nm.

To test the practicality of our strategy, we applied the sensor for Bcl-xL detection in 2% serum. We optimized the GO concentration in the range of 0–2 μg/mL in 2% serum system as described in a previous study [30]. As shown in Figure 6a, in the presence of Bcl-xL, the fluorescence intensity of TAM-PEP (R8) quenched by GO was increased to different degrees because of the different interaction strengths between the peptides and GO. Notably, the much smaller change in fluorescence intensity in the serum assay compared with that in the Tris-HCl buffer assay resulted from the background Raman scattering and fluorescent components in the serum [32]. There were various degrees of fluorescence change at different concentrations of GO (Figure S1), which reached the maximum value when the GO concentration was 0.25 μg/mL (Figure 6a). As a result, 0.25 μg/mL was considered as the optimized concentration of GO in 2% serum assay.

Figure 6b shows that the fluorescence intensity of TAM-PEP (R8) increased with the addition of Bcl-xL in 2% serum. The inset exhibited that there was good linear correlation between the fluorescence intensity and Bcl-xL concentrations in the range from 0 to 1.0 μM, suggesting that Bcl-xL was quantitatively detected by the peptide-GO sensing platform in 2% serum. These results demonstrated that this strategy could be used to detect Bcl-xL in serum.

### 3.5. Cell Imaging

The results of an investigation accessing the in vitro response show that the sensor can be used to detect Bcl-xL in living cells. The feasibility of utilizing the sensor for fluorescence imaging of intracellular Bcl-xL was explored next. For this purpose, we first conducted an MTT assay to test the cytotoxicity of GO, as shown in Figure S2, when the GO concentration was less than 10 μg/mL, there was almost no influence on cell viability. Next, we optimized the GO concentration in cell imaging. Hela cells were incubated with 1 μM TAM-PEP (R8) in growth media at 37 °C for 3 h and clear red intracellular fluorescence could be observed by confocal fluorescence microscopy (Figure S3a). In contrast, only weak fluorescence displayed in Figure S3b,c, which were incubated with the mixture of peptide (1 μM) and GO (5 μg/mL and 10 μg/mL) for 3 h. We chose 5 μg/mL GO in cell imaging, which had very low cytotoxicity. It is noteworthy that the concentration of GO is much lower than previous studies in cell imaging [16,17,31]. These results strongly indicated that the sensor permeates into cells and possesses a good stability in cells.

**Figure 7:**
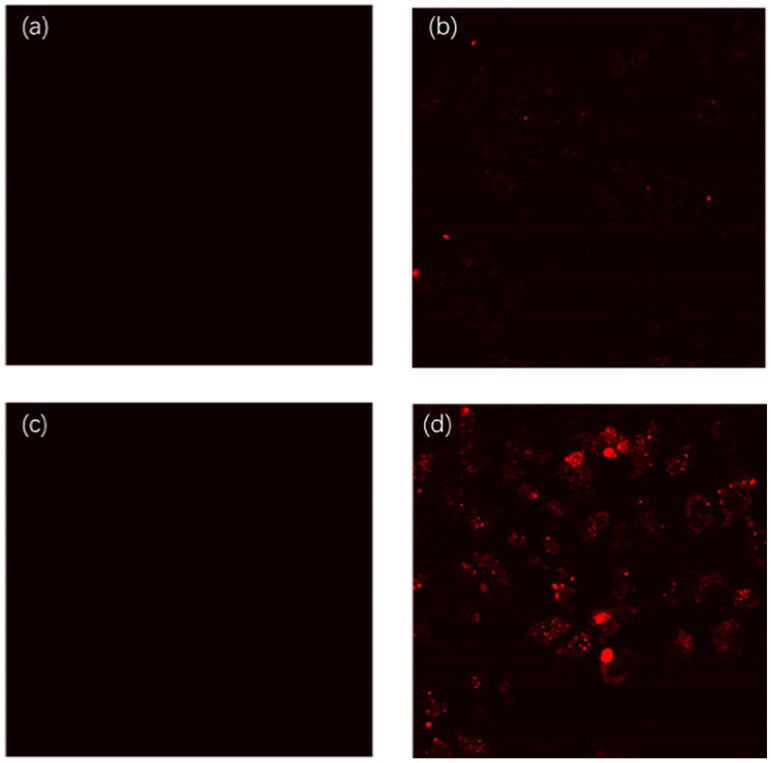
Fluorescence images of TAM-PEP (R8)/GO mixture in Hela cells and Bcl-xL overexpression cells. (**a**) Fluorescence images of only Hela cells; (**b**) Fluorescence images of TAM-PEP (R8)/GO mixture in Hela cells; (**c**) Fluorescence images of Bcl-xL overexpression cells; (**d**) Fluorescence images of TAM-PEP (R8)/GO mixture in Bcl-xL overexpression cells. Experiment conditions: TAM-R8-PEP: 1 μM; GO: 5 μg/mL; Excitation: 559 nm.

Providing a vivid contrast to Hela cells, we constructed the Bcl-xL overexpression cells which was certified through Western blotting. (Figure S4) Then, the TAM-PEP (R8)/GO mixture was co-incubated with Hela cells and Bcl-xL overexpression cells respectively for 3 h. As can be seen by viewing the confocal fluorescence microscope images shown in Figure 7, remarkable red intracellular fluorescence recovery could be monitored in Bcl-xL overexpression cells compared with the nature Hela cells due to the different amount of the Bcl-xL target. The large amount of Bcl-xL protein in the overexpression cells could recognize and bind with the peptide, leaving it from the GO surface and emitting the significant fluorescence. These results proved that our peptide-GO sensor could be applied for fluorescence imaging and detection of target in cells.

## 4. Conclusions

In conclusion, a highly sensitive and selective fluorescent turn-on assay for signaling target protein was developed by utilizing peptide-GO sensors. This peptide-GO sensor design consists of a fluorophore, a peptide containing eight arginine residues and peptide ligand that recognizes the target protein, and GO as a quencher. The feasibility of our design was confirmed by the sensitive and specific detection of Bcl-xL. By simply replacing the corresponding peptides of targets, this strategy can be easily generalized to other disease-related protein targets. Our sensor was proved to be stable and sensitive for the detection of the target proteins in buffer, 2% serum and living cells with much lower GO concentration compared to previous studies. Therefore, it can be applied for disease diagnosis, prognosis tracking and therapeutic evaluation.

## Supplementary Materials

The following are available online at http://www.mdpi.com/1424-8220/18/2/385/s1.

## References

[1] Tompa P. (2016). The principle of conformational signaling. Chem. Soc. Rev..

[2] Tang X.T., Sun M., Lu M.X., Du Y.Z. (2015). Expression patterns of five heat shock proteins in Sesamia inferens (Lepidoptera: Noctuidae) during heat stress. J. Asia Pac. Entomol..

[3] Jia X., Song T., Liu Y., Meng L., Mao X. (2017). An immunochromatographic assay for carcinoembryonic antigen on cotton thread using a composite of carbon nanotubes and gold nanoparticles as reporters. Anal. Chim. Acta.

[4] Pihikova D., Kasak P., Kubanikova P., Sokol R., Tkac J. (2016). Aberrant sialylation of a prostate-specific antigen: Electrochemical label-free glycoprofiling in prostate cancer serum samples. Anal. Chim. Acta.

[5] Li M., Shi Z., Fang C., Gao A., Li C.M., Yu L. (2016). Versatile microfluidic complement fixation test for disease biomarker detection. Anal. Chim. Acta.

[6] Wu L., Qu X. (2015). Cancer biomarker detection: Recent achievements and challenges. Chem. Soc. Rev..

[7] Saita T., Yamamoto Y., Hosoya K., Yamamoto Y., Kimura S., Narisawa Y., Shin M. (2017). An ultra-specific and sensitive sandwich ELISA for imatinib using two anti-imatinib antibodies. Anal. Chim. Acta.

[8] Han Z., Wang Y., Duan X. (2017). Biofunctional polyelectrolytes assembling on biosensors—A versatile surface coating method for protein detections. Anal. Chim. Acta.

[9] Li H., Hitchins V.M., Wickramasekara S. (2016). Rapid detection of bacterial endotoxins in ophthalmic viscosurgical device materials by direct analysis in real time mass spectrometry. Anal. Chim. Acta.

[10] Gao J., Ma H., Lv X., Yan T., Li N., Cao W., Wei Q. (2015). A novel electrochemical immunosensor using β-cyclodextrins functionalized silver supported adamantine-modified glucose oxidase as labels for ultrasensitive detection of alpha-fetoprotein. Anal. Chim. Acta.

[11] Peng W.P., Chou S.W., Patil A.A. (2014). Measuring masses of large biomolecules and bioparticles using mass spectrometric techniques. Analyst.

[12] Zhao M., Zhuo Y., Chai Y., Xiang Y., Liao N., Gui G., Yuan R. (2013). Dual signal amplification strategy for the fabrication of an ultrasensitive electrochemiluminescenct aptasensor. Analyst.

[13] Gao N., Gao F., He S., Zhu Q., Huang J., Tanaka H., Wang Q. (2017). Graphene oxide directed in-situ deposition of electroactive silver nanoparticles and its electrochemical sensing application for DNA analysis. Anal. Chim. Acta.

[14] Chullasat K., Nurerk P., Kanatharana P., Kueseng P., Sukchuay T., Bunkoed O. (2017). Hybrid monolith sorbent of polypyrrole-coated graphene oxide incorporated into a polyvinyl alcohol cryogel for extraction and enrichment of sulfonamides from water samples. Anal. Chim. Acta.

[15] Li S., Aphale A.N., Macwan I.G., Patra P.K., Gonzalez W.G., Miksovska J., Leblanc R.M. (2012). Graphene Oxide as a Quencher for Fluorescent Assay of Amino Acids, Peptides, and Proteins. ACS Appl. Mater. Interfaces.

[16] Feng D., Zhang Y., Feng T., Shi W., Li X., Ma H. (2011). A graphene oxide-peptide fluorescence sensor tailor-made for simple and sensitive detection of matrix metalloproteinase 2. Chem. Commun..

[17] Wang H., Zhang Q., Chu X., Chen T., Ge J., Yu R. (2011). Graphene oxide-peptide conjugate as an intracellular protease sensor for caspase-3 activation imaging in live cells. Angew. Chem..

[18] Wu D., Gao Y., Qi Y., Chen L., Ma Y., Li Y. (2014). Peptide-based cancer therapy: Opportunity and challenge. Cancer Lett..

[19] Zhang M., Yin B.C., Wang X.F., Ye B.C. (2011). Interaction of peptides with graphene oxide and its application for real-time monitoring of protease activity. Chem. Commun..

[20] Gu X., Yang G., Zhang G., Zhang D., Zhu D. (2011). A new fluorescence turn-on assay for trypsin and inhibitor screening based on graphene oxide. ACS Appl. Mater. Interfaces.

[21] Li D., Müller M.B., Gilje S., Kaner R.B., Wallace G.G. (2008). Processable aqueous dispersions of graphene nanosheets. Nat. Nanotechnol..

[22] Lerf A., He H., Forster M., Klinowski J. (1998). Structure of Graphite Oxide RevisitedII. J. Phys. Chem. B.

[23] Li X., Ding X., Fan J. (2015). Nicking endonuclease-assisted signal amplification of a split molecular aptamer beacon for biomolecule detection using graphene oxide as a sensing platform. Analyst.

[24] Zhang J., Tao M., Jin Y. (2014). An enzyme-aided amplification strategy for sensitive detection of DNA utilizing graphene oxide (GO) as a fluorescence quencher. Analyst.

[25] Dho S.H., Deverman B.E., Lapid C., Manson S.R., Gan L., Riehm J.J., Aurora R., Kwon K.S., Weintraub S.J. (2013). Control of cellular Bcl-xL levels by deamidation-regulated degradation. PLoS Biol..

[26] Yu F., Liang Z., Hu T., Xu S., Ding J., Chen K., Jiang H., Liu D. (2009). A conserved hydrophobic core at Bcl-xL mediates its structural stability and binding affinity with BH3-domain peptide of pro-apoptotic protein. Arch. Biochem. Biophys..

[27] Sun Z., Lu W., Tang Y., Zhang J., Chen J., Deng H., Li X., Liu J. (2007). Expression, purification and characterization of human urodilatin in *E. coli*. Protein Expr. Purif..

[28] Zheng J., Tan C., Xue P., Cao J., Liu F., Tan Y., Jiang Y. (2016). Proteolysis targeting peptide (PROTAP) strategy for protein ubiquitination and degradation. Biochem. Biophys. Res. Commun..

[29] Lv Y., Wu J., Wu P., Chen Y.Z., Tan Y., Tan C., Jiang Y. (2016). A sensitive polymeric dark quencher-based sensing platform for fluorescence “turn on” detection of proteins. RSC Adv..

[30] He Y., Lin Y., Tang H., Pang D. (2012). A graphene oxide-based fluorescent aptasensor for the turn-on detection of epithelial tumor marker mucin 1. Nanoscale.

[31] Li L., Feng J., Liu H., Li Q., Tong L., Tang B. (2016). Two-color Imaging of MicroRNA with Enzyme-Free Signal Amplification via Hybridization Chain Reactions in Living Cells. Chem. Sci..

[32] Jiang Y., Fang X., Bai C. (2004). Signaling Aptamer/Protein Binding by a Molecular Light Switch Complex. Anal. Chem..

