# Supplementary material for: A New Strategy Involving the Use of Peptides and Graphene Oxide for Fluorescence Turn-on Detection of Proteins
Source: Sensors (Basel). 2018 Jan 29;18(2):385. doi: 10.3390/s18020385 (PMC5855184; doi:10.3390/s18020385)

Supplementary Information

A New Strategy Involving the Use of Peptides and Graphene Oxide for Fluorescence Turn-on Detection of Proteins

Huan Shi[a,b, ‡], Bibo Zhang[a,b, ‡], Shuwen Liu[a,b], Chunyan Tan[a,b], Ying Tan[a,b,*], Yuyang Jiang[b,c]

a Department of Chemistry, Tsinghua University, Beijing 100084, P. R. China

b The State Key Laboratory Breeding Base-Shenzhen Key Laboratory of Chemical Biology, the Graduate School at Shenzhen, Tsinghua University, Shenzhen 518055, P. R. China.

c School of Pharmaceutical Sciences, Tsinghua University, Beijing 100084, P. R. China

‡ These authors contributed equally to this work.

* Correspondence: tan.ying@sz.tsinghua.edu.cn; Tel.: +86-0755-26036533

Table S1. Peptide probe sequences binding with Bcl-xL in the design.

No.	Sequence
TAM-PEP (R4)	TAM-RRRRNLWAAQRYGRELRRMSDKFVD
TAM-PEP (R6)	TAM-RRRRRRNLWAAQRYGRELRRMSDKFVD
TAM-PEP (R8)	TAM-RRRRRRRRNLWAAQRYGRELRRMSDKFVD

Figure Legends

Fig. S1 The Fluorescence intensity changes of GO-based aptasensor in the presence (red) and in the absence (black) of 5 μM Bcl-xL with the increasing amount of GO in the 2% serum. The inset is the recovery ratio of the fluorescence intensity of TAM-PEP (R8) at the emission of 580 nm with different GO concentration, where F is the fluorescence intensity with Bcl-xL (5 μM), F0 is the fluorescence intensity without Bcl-xL (5 μM). Experiment conditions: TAM-PEP (R8) 100 nM; Incubation time: 1 h; Room temperature. Excitation: 540 nm.

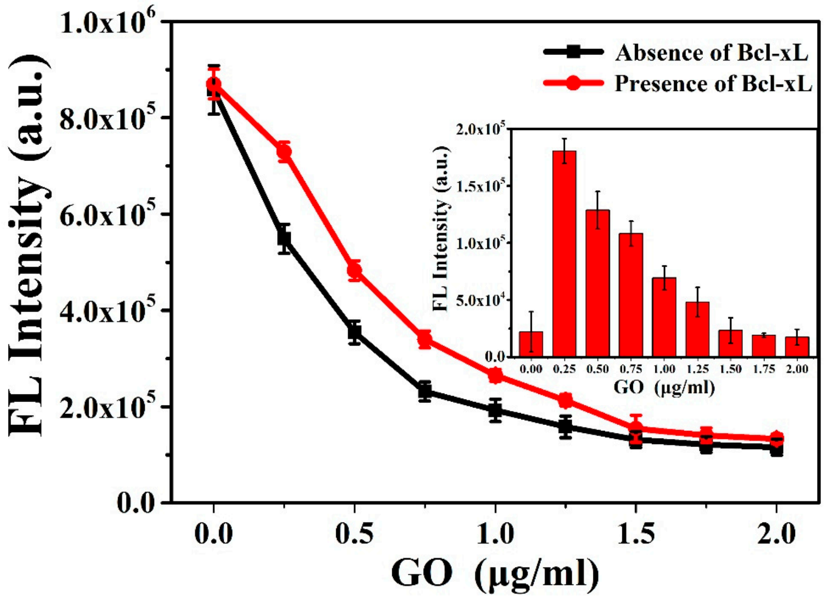

Fig. S2 Cytotoxicity induced by GO in Hela cells

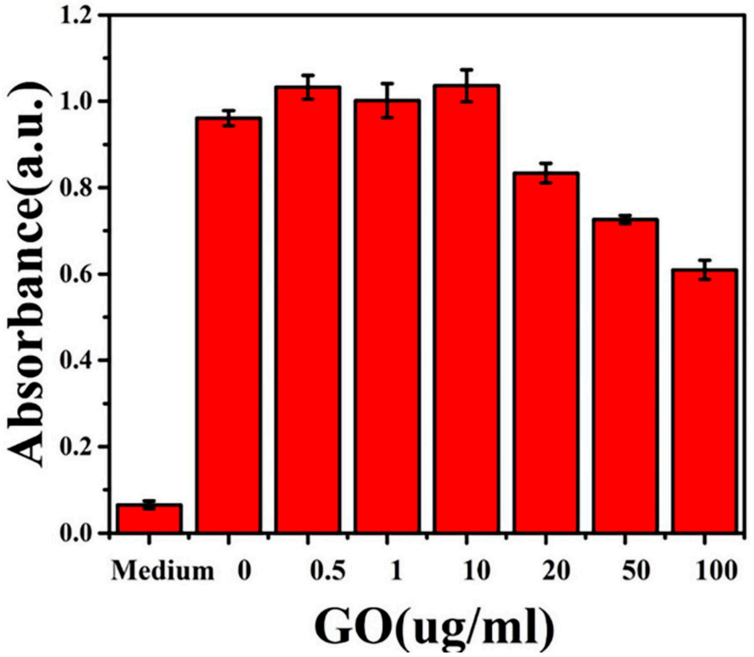

Fig. S3 (a) Fluorescence images of TAM-PEP (R8) in Hela cells. (b) Fluorescence images of TAM-PEP (R8)/GO mixture in Hela cells. GO: 5 μg mL^{-1} (c) Fluorescence images of TAM-PEP (R8)/GO mixture in Hela cells. GO: 10 μg mL^{-1} Experiment conditions: TAM-R8-PEP: 1 μM; Excitation: 540 nm.

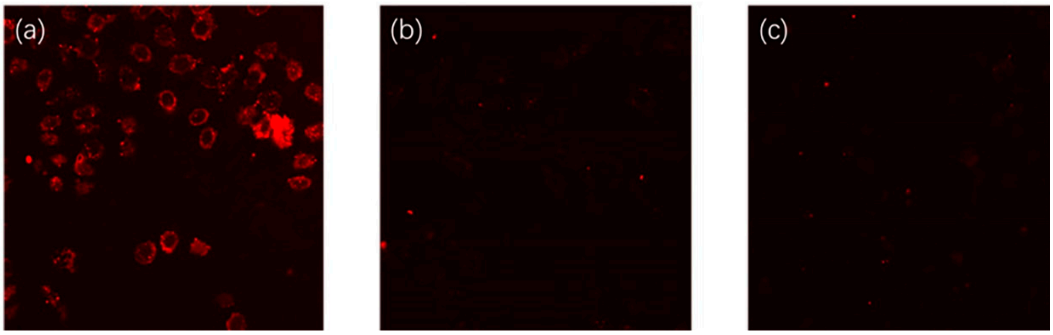

Fig. S4 Western blotting determination and quantification of proteins expression in untransfected Hela cells (A) and transfected Hela cells (B)

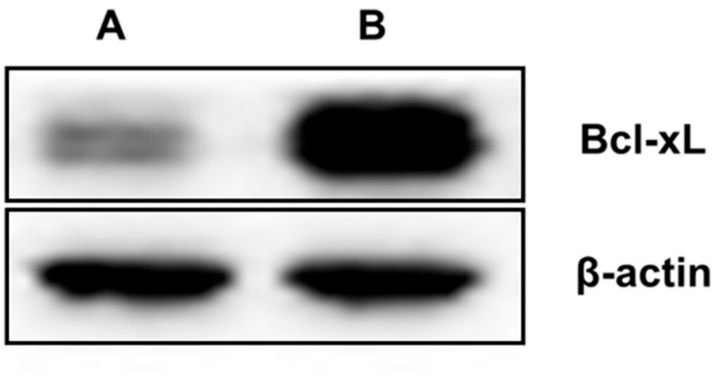

Supplement: Supplementary file 1 [file sensors-18-00385-s001.pdf]
